# In vivo confocal microscopic evaluation of patients with epidermolysis bullosa demonstrates severe loss of corneal nerves^☆^

**DOI:** 10.1016/j.ajoc.2026.102519

**Published:** 2026-02-05

**Authors:** Leyla Yavuz Saricay, Derya Goksu Fidan, Sara Galinko, Erin Huynh, Ariana Thompson, Vicki M. Chen, Pedram Hamrah

**Affiliations:** a Boston Children's Hospital, Harvard Medical School, Boston, MA, USA; b Boston Medical Center, Boston University School of Medicine, Boston, MA, USA; c Koc University, School of Medicine, Istanbul, Turkey; d Dana-Farber Cancer Institute, Harvard Medical School, Boston, MA, USA; e Pediatric Ophthalmology, New England Eye Center, Department of Ophthalmology, Tufts Medical Center, Tufts University School of Medicine, Boston, MA, USA; f The University of New Mexico, School of Medicine, University of New Mexico, USA; g U.S. Food and Drug Administration, Center for Devices and Radiological Health (CDRH), Division of Ophthalmic Devices, Boston, MA, USA; h USF Health, Department of Ophthalmology, University of South Florida, Tampa, FL, USA

**Keywords:** Epidermolysis bullosa, Neurotrophic keratitis, Corneal nerve loss, In vivo confocal microscopy

## Abstract

**Purpose:**

Epidermolysis bullosa (EB) is a rare inherited genetic disorder associated with severe ocular surface complications and progressive corneal scarring. This case series aimed to structurally characterize and quantitatively analyze central corneal subbasal nerve plexus loss using in vivo confocal microscopy (IVCM) in pediatric patients with recessive dystrophic epidermolysis bullosa (RDEB).

**Observations:**

This retrospective case series included five pediatric patients with RDEB presenting with ocular and systemic manifestations. In vivo confocal microscopy (IVCM) imaging showed severe central subbasal corneal nerve plexus loss in 85% of eyes, with a median total nerve density of 0 μm/mm^2^ (IQR: 0–5150.67), main nerve trunk density of 0 μm/mm^2^ (IQR: 0–2720.56), and branch nerve density of 0 μm/mm^2^ (IQR: 0–2769.22). Dendritiform reflective structures were qualitatively observed in multiple scans of eyes demonstrating marked central subbasal nerve loss.No immunophenotyping was performed. Corneal sensation testing was not performed due to severe ocular surface fragility and photophobia; therefore, no clinical diagnosis or staging of NK was inferred or concluded from this cohort.

**Conclusions and importance:**

This series identifies profound structural corneal denervation of the central subbasal plexus on IVCM in pediatric RDEB-associated ocular surface disease. The findings support future prospective studies investigating corneal nerve-regeneration and epithelial-stabilization frameworks to preserve long-term visual outcomes in children with RDEB.

## 1 Introduction

Epidermolysis bullosa (EB) is characterized by epithelial fragility and blistering of the skin, mucosa, and ocular surface.^1^^,^^2^ In the United States, hereditary EB has an incidence of 19.57 per 1 million live births.^3^ Even rarer, with an incidence of 1.35 per 1 million, the subgroup of recessive dystrophic EB (RDEB) is commonly associated with ocular manifestations of recurrent corneal erosions, persistent epithelial defects, ocular pain, scarring, and progressive vision loss.^3^^,^^4^ These problems typically arise after mild ocular trauma, such as rubbing an eyelash on the ocular surface.^5^^,^^6^ Genetic mutations result in structural and functional changes of corneal basement membrane proteins, particularly the anchoring fibrils that hold together epithelial layers in the cornea.^7^ Patients with RDEB have mutations in the *COL7A1* gene, which encodes collagen VII (CVII)—a key component of anchoring fibrils that connect the epidermis to the underlying basement membrane.^8^ In some cases, CVII may be produced in normal or near-normal amounts but is structurally abnormal or non-functional due to mutation.^9^ Typically, patients with RDEB present with severe pain alongside acute and recurrent abrasions or erosions. However, some patients with severe vision loss and active erosions only reported rare abrasions and remote pain episodes as infants.^6^ Pain intensity can fluctuate over time; however, in this series all five patients reported ocular pain or discomfort at presentation, which prompted detailed assessment of corneal nerves with IVCM.

Corneal nerve depletion can accompany chronic ocular surface injury in RDEB and is frequently underrecognized without targeted corneal nerve imaging such as IVCM. Corneal surface trauma can lead to persistent epithelial defects, corneal opacification, progressive stromal scarring, surface irregularity, induced refractive error, and deprivation amblyopia, as demonstrated extensively in NK.^10^ Similarly, patients with EB have recurrent painful ulcers with minor trauma or without known trauma which can result in cornea scarring and opacification.^11^ Therefore, without IVCM, reduced corneal nerve density and corneal neuropathy are frequently overlooked.^10^^,^^12^^,^^13^

Systematic investigation of the pathogenic relevance of corneal nerve loss in ocular RDEB is warranted, as the disease currently lacks disease-specific ocular therapies.^14^ Current management is based on intensive lubrication to reduce surface dryness and prevent abrasions.^14^ Although mechanically protective, lubrication is not biologically restorative and does not address the downstream consequences of disrupted nerve-dependent trophic signaling. NK remains the most extensively studied corneal denervation-associated epithelial injury model, demonstrating that loss of nerve-derived trophic signaling compromises epithelial integrity and delays corneal wound healing.^10^ These neuro-epithelial signaling interactions provide a mechanistic rationale for investigating similar trophic pathways in ocular RDEB, where profound central subbasal corneal nerve loss has been consistently visualized on IVCM in scarred corneas.

Accordingly, we hypothesize that structural corneal denervation may contribute to recurrent epithelial instability and impaired corneal repair in RDEB through disruption of nerve-dependent trophic support.

## 2 Materials and methods

### 2.1 Patients

These retrospective case presentations included five pediatric patients, aged 11 (n = 3), 14 (n = 1), and 18 years (n = 1); among them were three females and two males with confirmed diagnoses of RDEB, evaluated at the New England Eye Center (Tufts Medical Center, Tufts University School of Medicine, MA). Data extracted from the medical records included patient demographics, systemic medical history, ocular history, previous treatments, best-corrected visual acuity (BCVA), clinical findings, ocular and periocular involvement, comprehensive slit-lamp biomicroscopic evaluation, and laser in vivo confocal microscopy (IVCM, HRT3/RCM, Heidelberg Engineering GmBH, Heidelberg, Germany) results. Corneal nerve densities were analyzed from IVCM scans obtained at each patient's initial presentation.

The Tufts University Health Sciences Institutional Review Board approved this retrospective observational case series. The protocol conformed to the Declaration of Helsinki and adhered to the Health Insurance Portability and Accountability Act (HIPAA). Informed consent was not obtained because this was a retrospective chart review and was exempt from informed consent requirements.

### 2.2 In vivo confocal microscopy

IVCM is a standardized and safe procedure to obtain precise measurements of corneal morphology. At each patient's initial visit, IVCM (HRT3/RCM) of the central cornea was performed according to Cruzat et al.^15^ Images were taken with a 63 × objective immersion lens with 0.9 aperture, producing a 400 × 400 μm (160,000 μm^2^) image of the central cornea. In brief, topical 0.5% proparacaine hydrochloride (Alcaine; Novartis Ophthalmics) was administered to both eyes, followed by a drop of hydroxypropyl methylcellulose 2.5% (GenTeal Gel, Alcon, Fort Worth, TX) to increase optical coupling. A sterile, disposable polymethylmethacrylate cap was filled with hydroxypropyl methylcellulose 2.5% and applied to the outer surface of the lids.

Six to eight sequence scans were performed through the entire thickness of each central cornea, yielding approximately 50–100 images of the subbasal corneal nerve layer. This setup provided high-resolution en face images of the subbasal nerve plexus, measuring 400 × 400 μm with a lateral resolution of 1 μm/pixel.

### 2.3 Image analysis

Image selection and quantitative nerve analysis were performed as follows. A masked observer randomly selected three most representative images per eye from each set of 50–100 subbasal nerve plexus images. Despite significant ocular scarring and fibrosis, representative images were chosen based on optimal focus, complete nerve tracking, minimal motion artifacts, and consistency of the same layer from the central cornea. The same number of images (three per eye) was analyzed for all patients to ensure methodological consistency and reproducibility.

Nerve analysis was completed using the semi-automated tracing program NeuronJ, a plug-in for ImageJ (ImageJ, National Institutes of Health, Bethesda, Maryland). Main nerve trunks were defined as continuous nerve fibers that originated independently and did not branch from another nerve within the image frame. Branching nerves were defined as secondary fibers that diverged from these main trunks. The total lengths of main trunks and branching nerves were recorded separately.

Nerve density was calculated by dividing the total fiber length of each category by the fixed image area (0.16 mm^2^). Thus, main nerve density was defined as the total length of main trunks per unit area (μm/mm^2^), branch nerve density as the total length of branching nerves per unit area, and total nerve density as the cumulative length of all nerve fibers per unit area. To minimize intra-eye variability and reflect the non-normal distribution of nerve-density values, the median of the three analyzed images per eye was recorded as the representative nerve-density measurement for that eye.

For figure presentation, one representative image per eye was selected to illustrate corneal findings, while all quantitative data in the study were derived from the three-image median values. This approach provides a standardized and quantitative method for assessing the structural integrity of the corneal subbasal nerve plexus. As this was a retrospective study, analyses were based on clinical data and imaging collected by the treating physicians at the time of evaluation.

### 2.4 Analysis

Statistical analyses were performed with SPSS software version 22.0 (SPSS Inc., IBM, Chicago, IL, USA). Given the small sample size (5 patients, 10 eyes), only descriptive statistics were conducted. Nerve-density values and visual acuity were reported as median and interquartile range (IQR). Each eye was analyzed independently. Although this approach may introduce inter-eye correlation, both eyes were included to comprehensively capture the variability and extent of corneal nerve involvement in this rare disease cohort. Inter-eye comparisons were avoided to minimize potential statistical bias.

The Kolmogorov–Smirnov test was used to assess the normality of nerve-density distributions. Results indicated non-normal distributions for total nerve density (KS = 0.327, p = 0.0023), main trunk nerve density (KS = 0.445, p = 0.00000613), and branch nerve density (KS = 0.358, p = 0.00059); therefore, non-parametric descriptive statistics (median and IQR) were used throughout. Visual acuity was expressed in logarithm of the minimal angle of resolution (logMAR) units to align with published standards. Exploratory assessment of potential correlations between corneal nerve density parameters (total, main trunk, and branch) and clinical findings (age, best-corrected visual acuity, frequency of corneal abrasions, and presence of scarring or pannus) was considered; however, due to the small sample size and non-normal data distribution, formal correlation analyses were not performed.

## 3 Results

### 3.1 Cases

**Fig. 1:**
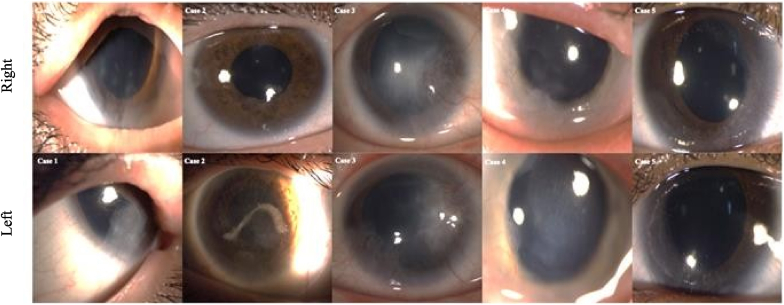
Slit-lamp biomicroscopic images of five patients with recessive dystrophic epidermolysis bullosa (RDEB). Right eyes are shown in the top row and left eyes in the bottom row. Corneas show epithelial irregularity, subepithelial haze or scarring, and peripheral superficial neovascularization (pannus) of varying severity. Images were obtained using a 63 × lens (0.9 aperture; 400 × 400 μm field).

The list of patient demographic features, history of ocular and systemic medical diseases, medications, clinical presentations (Table 1), and IVCM measurements are summarized in Table 2. Slit-lamp photos are shown in series in Fig. 1.

**Table 1:** Demographic and clinical characteristics of patients with recessive dystrophic epidermolysis bullosa.

Case	Age	Sex	Ocular History	Systemic/General Findings	logMAR BCVA		Corneal pannus (clock hours)		Ocular Medication	Systemic Medication
					OD	OS	OD	OS		
**1**	11	M	Recurrent corneal abrasion (2 × /week), corneal scarring, pseudopterygium OD, pannus OU, mild myopia, mild astigmatism	Hand/knee contracture, constipation, esophageal stricture, yearly esophageal dilation	0.4	0.5	0	1	Carboxymethylcellulose 1%, Artificial tear ointment	Diphenhydramine HCl, Melatonin
**2**	11	F	Corneal scarring, conjunctival inflammation, pannus, corneal blisters, repeated corneal abrasions, corneal scarring, ectropion, lagophthalmos, mild hyperopia, moderate-high astigmatism	Constipation	0.5	0.8	6	0	Artificial tearsArtificial tearointment, Carboxymethylcellulose 1%, Moxifloxacin, bandage contact lens	No known information
**3**	18	M	Pannus, pseudopterygium, corneal scarring, moderate myopia, moderate astigmatism	Hypotension	0.55	0.4	5	9	Artificial tear ointment, Loteprednol 0.2%, autologous serum tears, cyclosporine 0.09%, ofloxacin, bandage contact lens	Calcium, Zinc, Oxycodone, Lorazepam, Multivitamin
**4**	14	F	Mechanical ectropion, corneal scarring, repeated corneal abrasions, moderate hyperopia, moderate astigmatism	Chronic anemia, vitamin deficiency, esophageal stricture, yearly esophageal dilation	0.55	0.5	4	3	Artificial tears and ointment, Carboxymethylcellulose 1%	Doxycycline, Celebrex, Tylenol, Cetirizine
**5**	11	F	Dry eye disease, corneal scarring, pannus, repeated corneal abrasions, ghost vessels, deprivation amblyopia, high hyperopia, moderate astigmatism	Pre-term delivery, esophageal stricture, yearly esophageal dilation, G-tube, oral blisters, pseudosyndactyly of fingers and toes	0	0.4	9	6.5	Artificial tears, Carboxymethylcellulose 1%, Atropine 1% (for amblyopia penalization)	Cyproheptadine, Polyethylene glycol

**Abbreviations:** BCVA = Best-corrected visual acuity; HCl= Hydrochloride; OD = right eye; OS = left eye; OU = both eyes.

**Table 2:** Corneal nerve parameters measured by in vivo confocal microscopy in patients with recessive dystrophic epidermolysis bullosa (median [IQR]).

Case	Eye	Total Nerve Density (μm/mm^2^)	Main Trunk Nerve Density (μm/mm^2^)	Branch Nerve Density (μm/mm^2^)
		Median (IQR)	Median (IQR)	Median (IQR)
**1**	**OD**	0 (0-0)	0 (0-0)	0 (0-0)
	**OS**	0 (0-0)	0 (0-0)	0 (0-0)
**2**	**OD**	4455.25 (1669.94-6192.62)	0 (0-2506.44)	3686.19 (1669.94-4455.25)
	**OS**	0 (0-2021.69)	0 (0-0)	0 (0-2021.69)
**3**	**OD**	0 (0-0)	0 (0-0)	0 (0-0)
	**OS**	0 (0-0)	0 (0-0)	0 (0-0)
**4**	**OD**	4332.94 (1672.13-4426.44)	4332.94 (0-4426.44)	0 (0-1672.13)
	**OS**	0 (0-0)	0 (0-0)	0 (0-0)
**5**	**OD**	8776.06 (6189.25-10,974.62)	2443.56 (1809.25-5060.81)	5913.88 (4380.00-6332.44)
	**OS**	7236.94 (628.44-7467.88)	2997.56 (0-5384.69)	1852.25 (628.44-4470.31)

**Abbreviations:** OD = right eye; OS = left eye; IQR = interquartile range.

**Note:** Nerve density parameters are expressed in micrometers per square millimeter (μm/mm^2^). Values represent the median and interquartile range derived from in vivo confocal microscopy images of the central cornea.

### 3.2 Case #1

**Fig. 2:**
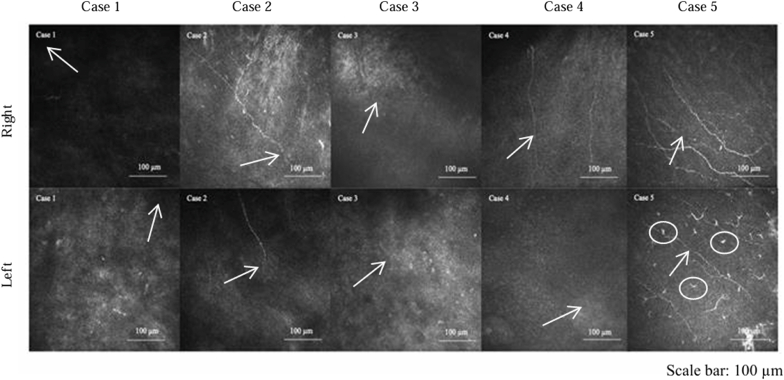
Representative in vivo confocal microscopy (IVCM) images of the central subbasal corneal nerve plexus in five patients with recessive dystrophic epidermolysis bullosa (RDEB), showing marked nerve loss with thin, fragmented, tortuous fibers and absence of the normal whorl-like pattern. Hyper-reflective dendritiform cells are visible in some eyes. (The arrows indicate nerve loss, with thin, fragmented, and tortuous fibers.The circle indicates hyper-reflective dendritiform cells).

An 11-year-old male with RDEB presented with frequent corneal abrasions, photophobia, and chronic pain worsened by blinking, resulting in the inability to open his eyes. The frequency of corneal abrasions increased from 1 to 2 episodes per month to 1–2 episodes per week over a 12-month period, treated with preservative-free artificial tears bilaterally, four times per day. History was significant for bilateral myopia, astigmatism, and pseudopterygium. The examination was positive for contractures of the hands and toes, multiple open wounds on the back, knees, elbows, and severe localized elbow tenderness. BCVA was 20/50 (right eye) and 20/60 (left eye). The right eye's slit-lamp examination revealed anterior stromal scarring, pannus with severe corneal vascularization, and prominent pseudopterygium (Fig. 1, Table 1). The left eye showed a pannus extending into the central region from the limbus (Fig. 1, Table 1). Both posterior poles were within normal limits. IVCM demonstrated sub-epithelial fibrosis and absence of corneal nerves with total nerve, main trunk nerve, and branch nerve densities in both eyes of 0 μm/mm^2^, 0 μm/mm^2^, 0 μm/mm^2^, respectively (Fig. 2, Table 2). Treatment for this patient was initiation of bandage contact lenses to reduce abrasion frequency, prophylactic topical antibiotics, and recommended carboxymethylcellulose 1% ophthalmic solution every 1-2 hours while awake.

### 3.3 Case #2

An 11-year-old female with RDEB presented with acute, bilateral corneal abrasions 2-4 times per month over 12 months. She was unable to open her eyes due to photophobia, chronic foreign body sensation, and pain upon blinking and awakening from sleep. Bandage contact lenses worsened her symptoms. Therefore, topical therapies were given as artificial tears and carboxymethylcellulose 1% bilaterally at least six times daily. She had a history of moderate bilateral astigmatism. Physical examination showed multiple open blisters on the arms and face, with contracture of both hands. BCVA was 20/60 (right eye) and 20/125 (left eye). Slit-lamp examination revealed symblepharon of the right upper lid to the temporal conjunctiva, anterior stromal scarring, and pannus (Table 1). The left eye showed opacity with irregular surface epithelium (Table 1). There was bilateral ectropion of the upper and lower lids, evidence of lagophthalmos, and a palpebral fissure opening of 3.0mm with eyelids slightly closed (Fig. 1). The posterior poles were within normal limits. IVCM demonstrated bilateral corneal scarring and severe corneal nerve loss shown by the median and IQR values of total nerve, main trunk nerve, and branch nerve densities in the right eye of 4455.25 (1669.94-6192.62) μm/mm^2^, 0 (0-2506.44) μm/mm^2^, 3686.19 (1669.94-4455.25) μm/mm^2^ and the left eye with 0 (0-2021.69) μm/mm^2^, 0 μm/mm^2^, 0 (0-2021.69) μm/mm^2^, respectively (Table 2). The patient was treated with a bandage contact lens in the left eye to promote the healing of irregular epithelium and fluctuating visual acuity, along with autologous serum tears bilaterally, eight times daily.

### 3.4 Case #3

An 18-year-old male with RDEB presented with an increasing frequency of corneal abrasions, a decline in visual acuity, and chronic and worsening ocular discomfort. The corneal abrasions lasted 1-2 days, twice a year, negatively impacting his daily living activities and school attendance. He reported bilateral and painful ocular infections at least four times per year, treated with one week of antibiotics. However, his chronic ocular discomfort increased and hindered blinking patterns. Ocular history was significant for dense pannus bilaterally, anisometropia, and pseudopterygium in the left eye, while systemic history included significant hypotension and repeated skin infections. Physical examination was notable for multiple open wounds on the back, knees, and elbows, along with healing blisters on the face with surrounding flaking skin. BCVA was 20/70 (right eye) and 20/50 (left eye). Slit-lamp examination of the right eye showed diffuse staining and pannus with active neovascularization inferiorly covering half the corneal surface extending into the visual axis (Fig. 1, Table 1). The left eye showed dense pannus extending into the visual axis, regressed vessels, prominent nasal pseudopterygium, an irregular corneal surface with minimal vessels, and corneal thinning paracentrally and inferiorly to the visual axis (Fig. 1, Table 1). The posterior poles were within normal limits. IVCM demonstrated subepithelial fibrosis and complete absence of corneal nerves bilaterally with total nerve, main trunk nerve, and branch nerve densities in both eyes of 0 μm/mm^2^, 0 μm/mm^2^, 0 μm/mm^2^, respectively (Fig. 2, Table 2). The patient was treated with bilateral autologous serum tears eight times daily, loteprednol etabonate ophthalmic solution twice daily, and increased lubricating ointment to 1-2 cm per night to cover the entire corneal surface.

### 3.5 Case #4

A 14-year-old female with RDEB presented with frequent, painful corneal abrasions occurring 1-2 days per episode, three times yearly. Therapy was ocular lubricant ointment at night with a foam dressing over both eyes to optimize moisture while sleeping. She had a history of worsening corneal scarring and 3-4 corneal abrasions per month from 6 to 8 years old. At presentation, this was managed with loteprednol etabonate ophthalmic solution but was discontinued due to increased intraocular pressure and cyclic vomiting coinciding with drops. Her history was also significant for bilateral hyperopia with astigmatism, esotropia, chronic anemia, multiple vitamin deficiencies, gastroesophageal reflux disease, and esophageal spasms. Physical examination was notable for multiple open healing wounds on the arms and face and bilateral 2+ mechanical ectropion of the upper lids. BCVA was 20/70 (right eye) and 20/60 (left eye). Slit-lamp examination of the right eye showed diffuse peripheral scarring, vascularized pannus, and diffuse scarring in the visual axis, causing an impaired red reflex after dilation (Fig. 1, Table 1). The left eye showed diffuse peripheral scarring with pannus (Fig. 1, Table 1). The posterior poles were within normal limits. IVCM demonstrated severe corneal nerve loss and scarring in the right eye and severe nerve loss in the right eye shown by the median and IQR values of total nerve, main trunk nerve, and branch nerve densities in the right eye of 4332.94 (1672.13-4426.44) μm/mm^2^, 4332.94 (0-4426.44) μm/mm^2^, 0 (0-1672.13) μm/mm^2^ and the left eye with 0 μm/mm^2^, 0 μm/mm^2^, 0 μm/mm^2^, respectively (Table 2). Treatment was altered to include preservative-free artificial tears 4-8 times daily.

### 3.6 Case #5

An 11-year-old female with RDEB presented with chronic eye pain, redness, and acute episodes of corneal abrasions occurring 1-2 times per year, lasting for a maximum of one day. Management was using artificial tears bilaterally two times a day. Her history included deprivation amblyopia in the left eye, peripheral corneal scarring, bilaterally keratoconjunctivitis sicca, and corneal blistering since age 2. Other significant histories were pruritus, oral blisters, stridor, dysphoria, chronic anemia, multiple vitamin deficiencies (primarily zinc and iron), esophageal spasms, and 90% daily nutrition acquired through a gastrostomy tube. Physical examination showed diffuse dark brown hyperpigmented macules and patches scattered over the trunk, extremities, and face, as well as blisters and erosions on the right medial thigh, upper back, bilateral knees, elbows, and shins. BCVA was 20/20 (right eye) and 20/50 (left eye). Slit-lamp examination of the right eye showed dense scarring in two areas, one with a pannus extending into the visual axis (Fig. 1, Table 1). The left eye showed one area of dense scarring and pannus with ghost vessels extending 2.0 mm into the visual axis (Fig. 1, Table 1). The posterior poles were within normal limits. IVCM demonstrated subepithelial scarring and severe corneal nerve loss shown by the median and IQR values of total nerve, main trunk nerve, and branch nerve densities in the right eye of 8776.06 (6189.25-10,974.62) μm/mm^2^, 2443.56 (1809.25-5060.81) μm/mm^2^, 5913.88 (4380.00-6332.44) μm/mm^2^ and the left eye with 7236.94 (628.44-7467.88) μm/mm^2^, 2997.56 (0-5384.69) μm/mm^2^, 1852.25 (628.44-4470.31) μm/mm^2^, respectively (Table 2). Treatment was altered to preservative-free artificial tears 4-8 times per day and additional lubricant eye ointment nightly. All five patients reported ocular pain or discomfort at the index visit (constant or episodic), as documented in the case narratives.

### 3.7 Correlations of corneal nerves to clinical parameters

In this case series of five patients with RDEB (median (IQR): 11 years (11-16)), IVCM revealed markedly reduced corneal nerve parameters. For each eye, total, main trunk, and branch nerve densities (μm/mm^2^) were calculated by summing nerve fiber lengths and dividing by the imaged area. Across all eyes, the median total nerve density was 0 μm/mm^2^ (IQR: 0–5150.67), main trunk nerve density was 0 μm/mm^2^ (IQR: 0–2720.56), and branch nerve density was 0 μm/mm^2^ (IQR: 0–2769.22). We compared these results to a separate study of 55 patients (mean age 13 years old) without EB used as healthy controls, and the total corneal nerve density was 24.8 ± 0.59 mm/mm^2^.^16^ No correlations were observed between nerve density and clinical parameters. In our cohort, exploratory assessment of potential correlations between corneal nerve parameters (total, main trunk, and branch) and clinical findings (age, best-corrected visual acuity, frequency of corneal abrasions, and presence of scarring or pannus) was considered; however, due to the small sample size and non-normal data distribution, formal correlation analyses were not performed.

In several IVCM scans, we noted the presence of bright dendritic-appearing reflective cells within the subbasal epithelial layer. While immunophenotyping was not performed, these structures are morphologically consistent with dendritic cells (DCs), bone marrow–derived antigen-presenting cells involved in corneal immune surveillance. Their presence was qualitatively recorded as present or absent in each eye.

## 4 Discussion

In this case series, pediatric patients with RDEB demonstrated marked structural corneal denervation of the central subbasal nerve plexus on IVCM compared with published normative data.^17^ Across all five cases, subepithelial fibrosis and epithelial irregularity were accompanied by profound loss of total, main trunk, and branch nerve fibers. In several scans, dendritic-appearing reflective cells were also noted within the subbasal layer, suggesting possible neuroimmune activity in regions of denervation.

Corneal nerves are essential for epithelial homeostasis, wound healing, and transparency.^18^^,^^19^ In RDEB, chronic epithelial microtrauma from recurrent corneal abrasions, persistent inflammation, and progressive scarring may contribute to secondary corneal nerve loss, followed by incomplete or aberrant nerve regeneration. However, the consistent observation of dendritic-appearing cells in areas of nerve loss indicates that inflammatory mechanisms may also contribute. Activated dendritic cells release cytokines such as IL-1β, IL-6, and TNF-α, which can impair axonal regrowth and promote neural damage.^20^, ^21^, ^22^, ^23^, ^24^ These findings support a dual mechanism—mechanical disruption from recurrent epithelial injury combined with local immune activation sustaining nerve degeneration. Similar patterns of decreased nerve density and increased dendritic cell infiltration have been reported in severe dry eye disease, herpetic keratitis, and ocular Graft-Versus-Host Disease (GVHD).^25^, ^26^, ^27^ The observation of dendritic-appearing cells in regions of marked nerve loss further supports the presence of local neuroimmune interactions. Prior studies have shown that dendritic cell density increases in ocular and systemic small fiber neuropathies, such as type 1 diabetes mellitus, where these cells secrete proinflammatory cytokines including TNF-α, IL-1β, and IL-6. Moreover, dendritic cells and corneal sensory nerves interact bidirectionally through neuropeptides such as substance P and CGRP, amplifying local inflammation and impairing axonal regeneration. In RDEB, such persistent inflammatory signaling may contribute to epithelial fragility, Schwann cell dysfunction,and impaired corneal nerve recovery.

Given the established role of chronic inflammation and extracellular matrix disruption in RDEB, these processes may create a microenvironment that impairs Schwann cell survival and axonal guidance, further limiting corneal nerve regeneration.

Although IVCM provides detailed structural information, it cannot evaluate corneal sensory function or substitute for clinical sensation testing required for NK diagnosis or staging. All patients reported ocular pain or discomfort at presentation, indicating that corneal sensory disturbance may be fluctuating, irregular, or partially preserved despite profound subbasal nerve loss, a phenomenon also described in non-EB corneal denervation and neuropathic ocular pain cohorts. This apparent paradox has been recognized in neurotrophic corneal neurodegeneration, where discomfort may persist even when sensitivity is reduced.In a recent study by Yavuz Saricay et al.,^20^ patients with stage 1 NK reported pain despite diminished sensation, with a mean OPAS score of 5.28, demonstrating the coexistence of pain with structurally or functionally impaired corneal nerves.Pain in denervated or regenerating corneas may arise from microneuroma formation, peripheral sensitization, and aberrant nerve regeneration, which can generate spontaneous ectopic activity and hyperalgesia. In addition, inflammatory mediators such as TNF-α, IL-1β, and IL-6 may sensitize trigeminal nociceptors, linking neuroimmune signaling to persistent ocular pain.^20^, ^21^, ^22^, ^23^, ^24^^,^^28^ Together, these findings suggest that corneal sensory-nerve degeneration and loss of nerve-derived trophic support may contribute to ocular surface instability in pediatric RDEB.

The coexistence of structural denervation and qualitatively observed dendritiform immune reflectivity underscores the mechanistic importance of epithelial trauma and local inflammation in corneal nerve degeneration in RDEB. This dual-pathogenic possibility supports the need for management strategies that prioritize epithelial protection and early inflammation control to limit progressive stromal scarring and secondary neural degeneration.Current treatments for EB-related ocular disease include lubrication, anti-inflammatory drops, and autologous serum.^5^^,^^29^, ^30^, ^31^, ^32^ Two patients in our cohort received serum tears with partial symptomatic improvement, and one patient was treated with loteprednol etabonate, suggesting benefit from anti-inflammatory and trophic support. Given the concurrent findings of nerve loss and dendritic cell infiltration, a combined therapeutic approach targeting both inflammation and nerve regeneration may have potential to reduce ocular surface complications, such as recurrent erosions, scarring, and progressive vision loss, though further studies are needed to confirm efficacy. The use of recombinant human nerve growth factor (rhNGF), approved for all stages of neurotrophic keratopathy,^33^, ^34^, ^35^, ^36^ may also be promising for EB-associated ocular surface disease. Early initiation of anti-inflammatory therapy could help reduce scarring and secondary nerve degeneration.

This study has several limitations. The small cohort and tertiary referral setting reduce external validity, and comparisons were made using previously published control data rather than contemporaneous matched controls. Quantitative corneal sensation testing was not performed, as severe photophobia and epithelial surface fragility limited patient tolerance and precluded reliable measurement. All analyses were retrospective and derived from clinical documentation recorded during routine patient care.

Across cases, IVCM revealed a consistent profile of severe corneal denervation and marked attenuation of the subbasal nerve plexus, accompanied by chronic epithelial pathology and infiltration of dendritic-appearing immune cells. These findings support a neuroimmune injury paradigm, in which recurrent epithelial trauma and persistent local inflammation may contribute to progressive corneal nerve depletion and impaired tissue repair. The current dataset cannot determine whether nerve loss reflects a primary neurodegenerative process or secondary denervation resulting from cumulative injury, fibrosis, and sustained immune activation.

Early subbasal nerve alterations detected on IVCM may still provide value for disease monitoring and severity-based risk stratification. Future investigations incorporating non-contact esthesiometry, longitudinal IVCM imaging, and tear-based inflammatory biomarker or cytokine profiling could help clarify the temporal relationship between inflammation and nerve loss. Detecting early corneal nerve changes may ultimately inform approaches aimed at limiting stromal scarring and preserving long-term visual function. Neuroregenerative therapies, including recombinant human nerve growth factor (rhNGF), together with early and sustained inflammation control, represent biologically plausible and promising directions for future translational and clinical research in RDEB-associated corneal disease.

## CRediT authorship contribution statement

**Leyla Yavuz Saricay:** Writing – review & editing, Writing – original draft, Visualization, Methodology, Data curation, Conceptualization. **Derya Goksu Fidan:** Writing – review & editing, Writing – original draft, Software, Resources, Methodology. **Sara Galinko:** Writing – original draft, Conceptualization. **Erin Huynh:** Writing – original draft, Conceptualization. **Ariana Thompson:** Conceptualization. **Vicki M. Chen:** Writing – review & editing, Conceptualization. **Pedram Hamrah:** Writing – review & editing.

## Disclaimer

The views expressed in this article are those of the authors and do not necessarily represent the views or policies of the U.S. Food and Drug Administration.

## Funding

This project was supported by Research to Prevent Blindness and the Massachusetts Lions Eye Research Fund.

Given the retrospective nature of the study, written consent to publish this case has not been obtained. This report does not contain any personal identifying information.

## Declaration of competing interest

The authors declare that they have no known competing financial interests or personal relationships that could have appeared to influence the work reported in this paper.

## Abbreviations

EB: Epidermolysis Bullosa
NK: Neurotrophic Keratopathy
CVII: Collagen VII/7
RDEB: Recessive Dystrophic Epidermolysis Bullosa
IVCM: In vivo Confocal Microscopy
BCVA: Best Corrected Visual Acuity
BCL: Bandage Contact Lens
TUBB3: Beta III Tubulin
NGF: Nerve Growth Factor
CGRP: Calcitonin Gene-Related Peptides
rhNGF: Recombinant Human Nerve Growth Factor
TNF-α: Tumor Necrosis Factor-alpha
IL-1β: Interleukin-1 beta
IL-6: Interleukin-6
COL7A1: Collagen type VII alpha 1 chain
